# Acne Vulgaris Associated with Metabolic Syndrome: A Three-Case Series Highlighting Pathophysiological Links and Therapeutic Challenges

**DOI:** 10.3390/diagnostics15162018

**Published:** 2025-08-12

**Authors:** Laura Maria Endres, Alexa Florina Bungau, Delia Mirela Tit, Gabriela S. Bungau, Ada Radu, Camelia Cristina Diaconu, Ruxandra Cristina Marin

**Affiliations:** 1 Doctoral School of Biomedical Sciences, Faculty of Medicine and Pharmacy, University of Oradea, 410087 Oradea, Romania; lendres@uoradea.ro (L.M.E.); gbungau@uoradea.ro (G.S.B.); adaradu@uoradea.ro (A.R.); marin.ruxandracristina@student.uoradea.ro (R.C.M.); 2 Department of Psycho-Neurosciences and Recovery, Faculty of Medicine and Pharmacy, University of Oradea, 410073 Oradea, Romania; 3 Department of Preclinical Disciplines, Faculty of Medicine and Pharmacy, University of Oradea, 410073 Oradea, Romania; 4 Department of Pharmacy, Faculty of Medicine and Pharmacy, University of Oradea, 410028 Oradea, Romania; 5 Internal Medicine Department, “Carol Davila” University of Medicine and Pharmacy, 050474 Bucharest, Romania; camelia.diaconu@umfcd.ro; 6 Internal Medicine Clinic, Clinical Emergency Hospital of Bucharest, 014461 Bucharest, Romania

**Keywords:** acne vulgaris, metabolic syndrome, insulin resistance, dyslipidemia, obesity, systemic inflammation, PCOS, adolescent health, dermatology, multidisciplinary care

## Abstract

**Background and Clinical Significance**: As a common inflammatory skin disorder, acne vulgaris is classically associated with sebum overproduction, follicular hyper keratinization, and *Cutibacterium acnes* proliferation. Emerging evidence suggests a link between severe or treatment-resistant acne and metabolic syndrome, characterized by central obesity, insulin resistance, dyslipidemia, and hypertension. This case series aims to explore the clinical overlap between acne and metabolic dysfunction and highlight the relevance of multidisciplinary evaluation. **Case Presentation**: Three patients with severe acne vulgaris and coexisting metabolic abnormalities were evaluated at a dermatology clinic in Oradea, Romania, between 2023 and 2024. Each patient underwent dermatologic examination, laboratory testing for metabolic and hormonal parameters, and individualized treatment. Management strategies included topical/systemic acne therapies combined with metabolic interventions (lifestyle modifications, metformin (in two cases), and lipid-lowering agents). Case 1 (female, 23) had obesity, insulin resistance, dyslipidemia, and polycystic ovary syndrome (PCOS). Case 2 (male, 19) presented with central obesity and atherogenic dyslipidemia. Case 3 (male, 18) showed insulin resistance, overweight status, and elevated inflammatory markers. All three showed suboptimal response to standard acne treatment. Adjunct metabolic management resulted in partial improvement within 3 months. One patient required isotretinoin after metabolic stabilization. **Conclusions**: These cases underscore the interplay between acne and metabolic dysfunction. Insulin resistance and systemic inflammation may contribute to therapeutic resistance in acne. Early recognition of metabolic syndrome features in patients with severe acne may improve treatment outcomes. Dermatologists should consider metabolic screening to guide comprehensive, multidisciplinary care.

## 1. Introduction

Acne vulgaris is a common inflammatory disease of the pilosebaceous unit that extends beyond adolescence into adulthood, exerting significant psychosocial and physical burdens when persistent or severe. Traditionally, acne development has been attributed to factors such as androgen-driven sebum overproduction, follicular hyper keratinization, *Cutibacterium acnes* colonization, and local inflammation [1].

In recent years, emerging evidence has pointed toward a more complex etiology, suggesting a significant association between acne vulgaris and metabolic syndrome (MetS), a cluster of metabolic abnormalities including central obesity, insulin resistance (IR), dyslipidemia, and hypertension. Although acne has long been regarded as a dermatologic condition, it now appears increasingly reflective of deeper systemic dysfunctions. Multiple clinical studies report a higher prevalence of IR and MetS in patients with acne, particularly in those with severe or persistent forms, compared to the general population [2]. Furthermore, studies demonstrated that males with acne had significantly higher post-load insulin and glucose at 60 min following an oral glucose tolerance test, findings suggesting early metabolic impairments among young adults with acne [3].

Acne and MetS are linked through shared pathophysiological pathways including mTORC1 hyperactivation, oxidative stress, and chronic low-grade inflammation [4]. These molecular imbalances create a biochemical environment conducive to acne development. Elevated insulin and insulin-like growth factor 1 (IGF-1) levels, as hallmarks of IR, activate mammalian target of rapamycin complex 1 (mTORC1) signaling, which in turn drives sebaceous lipogenesis and follicular hyper keratinization [5]. Consistently, a 2023 meta-analysis on oxidative stress reported that key features of MetS, including impaired glucose tolerance, hypertension, dyslipidemia, and obesity, are also frequently observed in individuals with moderate to severe acne [6].

Thus, IR continues to emerge as a central biological driver linking acne and MetS. A large-scale study of adult acne patients found IR markers in over 80% of cases, significantly higher than in controls [7]. Hyperinsulinemia resulting from IR can directly stimulate sebaceous glands and increase sebum production, aggravating acne lesion formation. Insulin and IGF-1 signaling promote sebocyte proliferation and androgen-mediated sebum secretion, creating a pro-acne environment. Furthermore, MetS is characterized by chronic low-grade systemic inflammation; elevated levels of pro-inflammatory cytokines (tumor necrosis factor alpha (TNF-α) and interleukin 6 (IL-6)) and markers such as C-reactive protein (CRP) have been observed in metabolically unhealthy patients [8,9].

Beyond insulin-related mechanisms, metabolic and endocrine comorbidities have also been linked to acne severity. Obesity and adiposity can worsen IR and inflammation, thereby potentially intensifying acne. This proposed link is further supported by epidemiological data. Multiple population-based studies have demonstrated that individuals with higher body mass index (BMI) or excess adiposity have an increased risk of developing acne and are more likely to present with moderate-to-severe forms of the condition across preadolescent, young adult, and adult populations [10,11].

Thyroid dysfunction and other hormonal imbalances have been noted in some patients with difficult acne. In this light, acne has even been proposed as a potential clinical warning sign of underlying metabolic conditions—central obesity, hypertension, dyslipidemia, and impaired glucose tolerance—in otherwise healthy-appearing individuals [12,13].

One cross-sectional analysis observed that patients with acne were more likely to fulfill MetS criteria and often displayed increased waist circumference and blood pressure compared to controls, even when differences in body mass index were slight [5].

Among the endocrine disturbances associated with MetS, hyperandrogenism (particularly in polycystic ovary syndrome (PCOS)) plays a significant role in acne pathophysiology. PCOS exemplifies the hormonal–metabolic overlap: excess circulating androgens (testosterone, dihydrotestosterone) stimulate sebaceous glands and upregulate lipid synthesis pathways in the skin, increasing sebum production and predisposing to comedonal and inflammatory lesions. Clinical data indicate that approximately 15–25% of women with PCOS exhibit acne as a symptom of their condition [14,15,16]. Conversely, hyperandrogenemia is present in up to 75–90% of women with PCOS, underlining how frequently androgen excess accompanies this syndrome. Additionally, IR enhances ovarian androgen production and reduces sex hormone-binding globulin, further raising free androgen levels and compounding acne severity [17].

Given all of this, it is widely recognized that individuals with severe or atypical acne may benefit from a screening for metabolic syndrome components. Dermatology practitioners are thus urged to maintain a high index of suspicion: what presents as resistant acne on the skin might be a clue to an insulin resistant (IR) state or other metabolic issues beneath. This paradigm shift toward a broader multidisciplinary diagnostic approach, looking beyond the skin and incorporating metabolic screening and endocrine evaluation into the care of difficult acne cases. For example, measuring waist circumference, fasting glucose, lipid profile, and insulin levels (or Homeostasis Model Assessment for Insulin Resistance (HOMA-IR)) in select acne patients can unmask early IR or dyslipidemia, enabling timely lifestyle or therapeutic interventions to mitigate progression to diabetes or cardiovascular disease [18].

Such a comprehensive approach aligns with the principles of precision medicine in dermatology. Effective acne management now often demands collaboration across specialties. Dermatologists, endocrinologists, and even nutritionists or cardiologists may need to work in concert to fully assess and treat the patient. Interdisciplinary coordination ensures that underlying conditions like PCOS, metabolic syndrome, or thyroid disorders are identified and managed alongside the skin disease [12,19]. Advancing care in inflammatory skin conditions influenced by metabolic dysfunction will require close collaboration between dermatology and other fields (endocrinology, microbiology), ultimately bridging systemic health and cutaneous treatment [20].

Considering the above insights, the present case series illustrates clinical patterns suggestive of a link between acne vulgaris and MetS, as observed in three patients with overlapping dermatological and metabolic features. By presenting these cases, we highlight potential shared pathophysiological mechanisms and therapeutic challenges and underscore the importance of considering metabolic evaluation not only in the management but also in the diagnostic assessment of patients with severe, atypical, or treatment-resistant acne.

## 2. Materials and Methods

This case series includes three patients with acne vulgaris who also fulfilled the criteria for MetS. They were evaluated sequentially at a private dermatology practice (Pelican Hospital, Oradea, Romania) between 2023 and 2024. All patients provided informed consent for their data and for images to be used in research and publication. Ethical approval was obtained from the Pelican Hospital Scientific Research Ethics Committee (Decision no. 41, dated 9 January 2023), and this study adhered to the principles of the Declaration of Helsinki.

For each patient, a detailed medical history was taken, including dermatologic history and systemic comorbidities. A thorough dermatological examination was performed, documenting the type, distribution, and severity of acne lesions [10].

Clinical photographs were taken at baseline and follow-up visits (approximately at 2 or 3 months), and all the images belong to the first author’s personal archive. The severity of acne was assessed based on lesion counts and qualitative grading (nodulocystic vs. inflammatory vs. comedonal predominance).

Metabolic and endocrine evaluations were conducted for each case. Anthropometric measurements included body mass index (BMI) and waist circumference. Blood pressure was measured to screen for hypertension. Laboratory investigations included fasting glucose and insulin (with calculation of the HOMA-IR, glycosylated hemoglobin (HbA1c), lipid profile (triglycerides, LDL, HDL cholesterol), and in relevant cases, hormonal panels (including total testosterone and, for the female patient, serum prolactin and ovarian ultrasound). Insulin resistance was defined as HOMA-IR >2.0–2.5 (varying by case and reference range). MetS was defined according to the joint International Diabetes Federation and American Heart Association/National Heart, Lung, and Blood Institute criteria (three or more of the following: central obesity by waist circumference, elevated fasting glucose, high triglycerides, low HDL, elevated blood pressure) [21].

Management plans were multidisciplinary. All patients received dermatologic therapy appropriate for severe acne, as well as interventions for metabolic abnormalities. Dermatologic treatments were tailored to each case but generally included systemic antibiotics and/or retinoids, along with a combination of topical agents (retinoids, benzoyl peroxide, antibiotics, etc.). Adjunctive dermatologic procedures (comedown extractions, chemical peels) were employed as needed. Simultaneously, patients received advice on diet and lifestyle changes aimed at weight reduction and improved insulin sensitivity (e.g., low glycemic-load diet and regular exercise). Drugs such as metformin (for insulin resistance) or statins (for dyslipidemia) were initiated in collaboration with primary care or endocrinology.

Therapeutic response was monitored, and at each follow-up, acne lesion counts and subjective improvements were noted. We defined a partial response as a noticeable reduction in inflammatory lesions without full clearance, and we noted the pace of improvement relative to typical expectations from standard acne therapy. Therapy switch to oral isotretinoin was considered once metabolic parameters (especially lipids) were better controlled and if initial therapies were insufficient.

## 3. Results

### 3.1. Case 1. Severe Acne in a Young Woman with PCOS, Microprolactinoma, Metabolic Syndrome, and Endocrine History

A 23-year-old female presented with a 5-year history of treatment-resistant acne vulgaris, predominantly affecting her face. Her medical history was notable for two major endocrine disorders: PCOS diagnosed three years prior and a microprolactinoma (pituitary adenoma) diagnosed in 2018. Both conditions were associated with clinical hyperandrogenism, evidenced by moderate hirsutism since prepuberty and persistent acne, as well as chronic hyperprolactinemia. She had no family history of acne. She was undergoing endocrine therapy with combined oral contraceptives (COCs) (ethinyl estradiol + drospirenone) to regulate her menstrual cycles and cabergoline, a dopamine agonist, to treat hyperprolactinemia. The patient also displayed central (abdominal) obesity, though no acanthosis nigricans was observed.

#### 3.1.1. Dermatologic Examination

**Figure 1:**
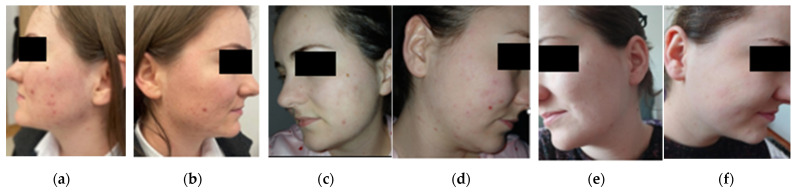
Severe nodulocystic acne with numerous comedowns, inflammatory lesions, and early scarring at baseline (**a**,**b**). At 2 months, marked reduction in cysts and nodules with residual erythema and emerging atrophic scars (**c**,**d**). By 6 months, inflammation largely resolved; mild papules, reduced seborrhea, and clearer scarring remain (**e**,**f**).

Cutaneous examination revealed multiple polymorphic acne lesions on the face, including open and closed comedowns, inflammatory papules and pustules, and several deep-seated nodules and cysts, especially on the cheeks and jawline. The facial skin was markedly seborrheic, with enlarged pores and a greasy appearance. Atrophic acne scars were present, suggesting recurrent flares and inadequate control over time (Figure 1a,b). Mild hirsutism (terminal hair on the upper lip and chin) was also noted. The patient had an android body habitus, with a protuberant abdomen relative to hip girth.

#### 3.1.2. Metabolic and Endocrine Profile

Biochemical evaluation confirmed the presence of IR and dyslipidemia, meeting the criteria for MetS. Anthropometric and laboratory data for Case 1 are summarized in Table 1, alongside their clinical interpretations.

#### 3.1.3. Final Diagnosis and Multidisciplinary Treatment Strategy

The patient was diagnosed with moderate-to-severe acne vulgaris, PCOS characterized by both biochemical and clinical hyperandrogenism, a microprolactinoma with chronic hyperprolactinemia, mixed dyslipidemia, and obesity with insulin resistance consistent with MetS. Moderate hirsutism was also present, further supporting the diagnosis of hyperandrogenic states. In response, a comprehensive, multidisciplinary treatment plan was implemented, targeting cutaneous inflammation, hormonal dysregulation, and underlying metabolic dysfunction through an integrated therapeutic approach.

Table 2 outlines the individualized therapeutic plan, personalized for Case 1, detailing the category of intervention, specific agents used, and their corresponding clinical objectives.

#### 3.1.4. Clinical Response and Follow-Up

After two months of therapy, partial clinical improvement was documented. The number of inflammatory lesions had decreased, and no new nodules developed. Seborrhea was still present but less pronounced. The overall improvement was slower compared to typical acne patients without metabolic disorders, suggesting that underlying IR and hormonal imbalances were modulating the therapeutic response (Figure 1c,d). Given her persistent dyslipidemia, oral isotretinoin was deferred until better lipid control could be achieved. At the 6-month follow-up, slight improvements in weight and metabolic parameters were noted, and a low-dose isotretinoin regimen was being considered under close metabolic monitoring (Figure 1e,f).

### 3.2. Case 2. Severe Nodulocystic Acne in a Male Adolescent with Metabolic Syndrome and Dyslipidemia Clinical Background

A 19-year and 5-month-old male presented with chronic, severe facial acne of approximately two years’ duration. His condition had proven refractory to various over-the-counter topical treatments (cleansers and creams) with minimal effect. Although he denied any family history of acne, his medical history revealed moderate central obesity (BMI ~30.2 kg/m^2^; weight 115 kg, height 1.96 m) and dyslipidemia, both detected on routine screening. He did not report any other chronic medical conditions. His body fat distribution was clearly central, with a protuberant abdomen, but no evidence of acanthosis nigricans was noted on examination.

#### 3.2.1. Dermatologic Examination

**Figure 2:**
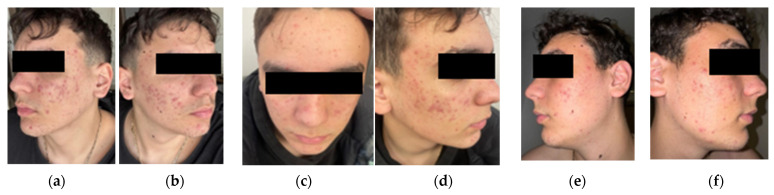
Initial presentation with numerous inflammatory papules, cysts, comedowns, and early scarring, especially on the malar and mandibular areas (**a**,**b**). At 2 months, reduced erythema and fewer active pustules and cysts, with no new nodules and moderate comedonal improvement (**c**,**d**). By 3 months, further decrease in inflammatory lesions and oiliness; persistent scarring and post-inflammatory changes are visible, but overall skin condition continues to improve (**e**,**f**).

Cutaneous evaluation revealed severe nodulocystic acne, with numerous closed and open comedowns, widespread erythematous papules and pustules, and deep, painful cystic nodules, particularly in the malar, chin, and jawline areas. Post-acne atrophic scars and hyperpigmented macules were visible, indicating multiple past inflammatory episodes. The skin was excessively oily with many congested follicles. No other skin abnormalities were found (Figure 2a,b).

#### 3.2.2. Metabolic and Endocrine Profile

Laboratory evaluation confirmed the presence of MetS. The HOMA-IR index exceeded the normal cutoff (3.57). Fasting glucose was mildly elevated (105 mg/dL), and while HbA1c was not obtained, his glycemia was at a threshold warranting lifestyle and dietary intervention.

To further characterize the patient’s metabolic and endocrine profile, a series of laboratory and anthropometric assessments were conducted. The findings confirmed the presence of multiple components of MetS, including IR, dyslipidemia, central obesity, and elevated blood pressure. Table 3 summarizes the key metabolic and hormonal parameters for Case 2, alongside their reference ranges and clinical interpretation.

#### 3.2.3. Final Diagnosis and Multidisciplinary Treatment Strategy

Considering the patient’s complex presentation, an individualized therapeutic strategy was developed, integrating both dermatologic and metabolic interventions. The approach emphasized initial inflammation control through topical and systemic antibiotics, supported by lifestyle and dietary adjustments to address insulin resistance and dyslipidemia. Table 4 outlines the complete therapeutic regimen applied in Case 2, detailing intervention categories, specific agents or strategies, and their corresponding clinical purposes.

#### 3.2.4. Clinical Response and Follow-Up

At the 2-month follow-up, the patient showed modest clinical improvement. The number of inflammatory lesions (papules and pustules) had slightly decreased, with reduced tenderness and no new nodular eruptions. Seborrhea was persistent, but skin oiliness was marginally reduced (Figure 2c,d). While some progress was made, the response to therapy was slower than typical for adolescents without metabolic complications.

The patient struggled with weight loss, highlighting the challenge of adherence to lifestyle interventions. However, laboratory monitoring showed gradual improvements in lipid profile, supported by statin therapy. By the 3-month mark, LDL and triglycerides had begun to normalize. Clinically, further improvement was noted, a reduction in the number and size of inflammatory lesions was visible, particularly on the lateral cheeks, with fewer new eruptions and a more stable cutaneous appearance (Figure 2e,f). Pending continued improvement, oral isotretinoin was planned as a next step, given its high efficacy in nodulocystic acne. However, treatment would be carefully monitored due to metabolic comorbidities.

### 3.3. Case 3. Severe Nodulocystic Acne with Early Conglobata Features in an Overweight Male with Dysmetabolic Profile Clinical Background

An 18-year-old male presented with a 1-year history of rapidly worsening, severe acne. He denied any family history of acne or metabolic disorders. While no prior metabolic condition had been formally diagnosed, his BMI was in the overweight range (~29.4 kg/m^2^), raising suspicion for an underlying dysmetabolic state. His acne had not responded to various pharmacy-obtained topical treatments.

#### 3.3.1. Dermatologic Examination

The patient had very severe, polymorphic acne, involving the face, upper neck, and upper back. The face was densely populated with closed and open comedowns, numerous inflamed papules and pustules, and several large, painful nodules and cysts, primarily on the cheeks, jawline, and chin. Marked erythema and edema surrounded many lesions.

**Figure 3:**
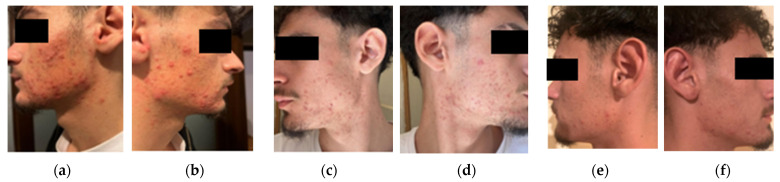
Initial presentation of severe acne with widespread papules, cysts, comedowns, and marked seborrhea; scarring and neck involvement reflect chronic inflammation (**a**,**b**). At 6 weeks, clear reduction in pustules and cysts, improved seborrhea, and no new nodules; early scars and post-inflammatory changes persist (**c**,**d**). By 3 months, further lesion reduction, improved skin texture, and continued fading of inflammatory signs, indicating sustained therapeutic response (**e**,**f**).

Acne also extended to the upper back, where a few large cysts and clusters of confluent pustules were observed, especially between the scapulae. These features were consistent with an early presentation of acne conglobata, a severe subtype of nodulocystic acne. Despite the relatively short history, atrophic scarring and post-inflammatory hyperpigmentation (PIH) were already present on the face and back. The skin was extremely oily, though no acanthosis nigricans was noted (Figure 3a,b).

#### 3.3.2. Metabolic and Endocrine Profile

Given the patient’s severe acne and body habitus, a metabolic and inflammatory evaluation was performed. The results revealed multiple abnormalities, including IR, dyslipidemia, and elevated inflammatory markers. These findings supported a diagnosis of a dysmetabolic profile, despite no prior formal diagnosis of metabolic syndrome. Table 5 presents a summary of the metabolic and endocrine parameters obtained in Case 3, along with clinical interpretations. These findings met at least three MetS criteria: central adiposity, high triglycerides, and low HDL, along with insulin resistance.

#### 3.3.3. Final Diagnosis and Multidisciplinary Treatment Strategy

The patient was diagnosed with severe nodulocystic acne vulgaris, exhibiting features approaching acne conglobata, in the context of a dysmetabolic profile characterized by insulin resistance, atherogenic dyslipidemia, and overweight. Laboratory evaluation also revealed low-grade systemic inflammation, which may have contributed to the severity and persistence of the acne. Given the high risk of scarring and the significant inflammatory burden, a prompt and aggressive treatment approach was adopted. Initial therapy included oral minocycline 100 mg/day, chosen for its anti-inflammatory properties and favorable dermal penetration. Topical agents consisted of adapalene 0.1% gel at night, benzoyl peroxide 5% gel in the morning, azelaic acid 20% cream applied to inflamed areas, and erythromycin cream applied twice daily to reduce bacterial colonization. Despite adherence, the patient continued to develop new cystic lesions after four weeks, prompting the initiation of oral isotretinoin at approximately 0.5 mg/kg/day, following baseline laboratory assessment and informed consent. In parallel, metabolic management efforts were intensified to address the underlying dysregulation contributing to the dermatologic presentation. Table 6 outlines the comprehensive therapeutic regimen applied in Case 3, including pharmacologic, lifestyle, and monitoring components, along with their corresponding clinical objectives.

#### 3.3.4. Clinical Response and Follow-Up

By week six of combined dermatologic and metabolic therapy, the patient exhibited early signs of improvement, including the cessation of new nodular lesion development, reduction in pustular activity, and noticeably diminished seborrhea and oiliness (Figure 3c,d). By the end of two months, more substantial progress was observed: there was a marked reduction in the number and size of existing pustules and nodules (Figure 3e,f), along with mild weight loss (approximately 5 kg) and early improvements in lipid parameters. Importantly, no significant adverse effects related to isotretinoin were reported, potentially due to the insulin-sensitizing effects of concurrent metformin use. Based on this favorable response, the therapeutic plan included continuation of isotretinoin for a full six- to eight-month course, accompanied by ongoing metabolic monitoring.

This case highlights the role of systemic inflammation and insulin resistance in sustaining severe, treatment-resistant acne. The delayed response to initial therapy underscores the importance of addressing underlying metabolic dysfunction. A combined approach targeting both cutaneous inflammation and metabolic imbalance was essential for early disease control and improved tolerability of systemic retinoid therapy.

### 3.4. Comparative Overview of the Three Cases

Table 7 provides a comparative summary of the three presented cases, highlighting both shared and distinct features in terms of clinical presentation, metabolic status, therapeutic strategy, and early treatment response. Despite differing in age and acne subtype, all patients demonstrated elements of metabolic dysfunction such as IR, dyslipidemia, and central adiposity, which could have influenced both the severity of their acne and the rate of therapeutic response. These observations underscore the importance of addressing systemic metabolic imbalances alongside standard dermatologic treatment, particularly in patients with severe or refractory acne.

## 4. Discussion

While acne remains one of the most frequent dermatologic conditions seen in clinical practice, not all cases respond predictably to standard topical or systemic therapies. This case series reinforces the emerging paradigm that acne vulgaris, especially in its severe, treatment-resistant, or atypical forms, may be a cutaneous manifestation of underlying systemic disorders, most frequently, MetS and endocrine dysregulation. Across all three cases presented, comprehensive diagnostic evaluations uncovered consistent metabolic abnormalities, including IR, dyslipidemia, central obesity, and in one case, hyperandrogenism from PCOS. These findings challenge the traditional notion of acne as a disease of adolescence or isolated sebaceous gland dysfunction and highlight the value of integrating clinical dermatology with metabolic and endocrinologic diagnostics [5,22]. Although all three patients shared the common denominator of severe acne and MetS features, their diagnostic profiles, comorbidities, and treatment responses illustrate critical nuances in managing acne within a precision dermatology framework.

Case 1, a young woman with a well-documented history of PCOS and a prolactin-secreting pituitary adenoma, demonstrates the compounded effect of dual endocrine disorders on skin pathology. Her acne was not only severe and persistent but also resistant to monotherapy, and her hormonal milieu, marked by hyperandrogenism and hyperprolactinemia, provided a pathophysiological backdrop that needed a layered treatment strategy. The addition of metformin, in addition to her existing endocrine therapy (COCs and cabergoline), was essential in modulating both IR and ovarian androgen production. This patient exemplifies how endocrine dysfunction can underlie both the etiology and treatment resistance of acne and underscores the need to integrate reproductive endocrinology into the dermatologic workup, especially in women presenting with features of hyperandrogenism or irregular menses.

In contrast, Case 2 represents a male adolescent with nodulocystic acne and previously undiagnosed metabolic dysfunction. Despite lacking endocrine disorders, he exhibited clear signs of MetS (central obesity, dyslipidemia, and IR), which appeared to blunt the efficacy of standard dermatologic treatments. His response to doxycycline and topical therapy was suboptimal, and isotretinoin initiation had to be postponed due to atherogenic lipid levels. This case illustrates the diagnostic value of metabolic screening in young male acne patients who may not otherwise present with classic signs of endocrine disease. Importantly, the multidisciplinary approach, combining dermatology with primary care and nutritional intervention, allowed for risk factor mitigation prior to initiating isotretinoin, highlighting the importance of lipid management in acne patients with dysmetabolic profiles. It also reflects how metabolic health directly impacts therapeutic timing and drug selection, especially in patients for whom systemic treatments carry metabolic side effects.

Case 3 introduces a distinct diagnostic scenario: a young man with explosive-onset acne of conglobata-like severity, coupled with inflammatory markers and borderline metabolic dysfunction. Unlike Case 2, his BMI was slightly lower, yet he already displayed multiple MetS criteria, including elevated triglycerides, IR, and an increased hs-CRP level. Notably, this patient also had a positive ASLO titer, raising the possibility of post-infectious or immune-mediated inflammatory amplification. His clinical picture aligned with recent studies suggesting that early, intense systemic inflammation, even in metabolically borderline individuals, may be sufficient to trigger or intensify acne conglobata-like features [23].

His case serves as a reminder that even in the absence of formal MetS diagnosis, the combination of IR and systemic inflammation can drive unusually aggressive acne phenotypes. Furthermore, this patient’s partial response only emerged after the introduction of metformin and lifestyle changes, which seemed to enhance the tolerability and efficacy of isotretinoin. This underscores the concept of “metabolic priming”—addressing systemic drivers before initiating definitive dermatologic therapy to optimize outcomes [24].

When analyzing the three cases comparatively, several critical diagnostic and therapeutic insights emerge, offering valuable implications for clinical practice. First, the timing of diagnosis played an important role in guiding management. In Case 1, the patient had established diagnoses of PCOS and a prolactin-secreting pituitary adenoma, which enabled the early initiation of targeted hormonal and metabolic therapies. In contrast, Cases 2 and 3 required comprehensive new investigations to uncover previously unrecognized metabolic dysfunctions. This discrepancy underscores the importance of early, multidisciplinary evaluation in patients with severe or atypical acne, particularly when clinical features suggest possible endocrine or metabolic involvement.

Second, the cases reveal how metabolic heterogeneity shapes the clinical expression and progression of acne. While all three individuals met the criteria for MetS, the dominant pathophysiological drivers varied. Case 1 reflected an endocrine-dominant pattern, Case 2 aligned with a classic dysmetabolic profile, and Case 3 exhibited a more inflammatory-metabolic phenotype. These distinctions likely contributed to differences in acne presentation, ranging from nodulocystic forms to conglobata-like patterns, and also influenced the rate and depth of the therapeutic response. This observation reinforces the concept that acne, particularly in its severe forms, is not a uniform condition but rather a dermatologic endpoint of diverse systemic imbalances.

Third, the use of diagnostic biomarkers proved instrumental in refining clinical decisions. The integration of laboratory assessments, such as HOMA-IR for IR, hs-CRP for systemic inflammation, lipid profiles, and hormone panels, provided clarity on systemic contributors and enabled more informed treatment planning. In some cases, these results prompted therapeutic adjustments, such as delaying isotretinoin in the context of hyperlipidemia or initiating metformin to address insulin resistance. This precision-guided approach is increasingly supported by recent literature. Studies demonstrated that combining hormonal and metabolic biomarkers with clinical acne scoring improved prediction of disease severity and allowed for more individualized treatment algorithms [25].

These objective diagnostics proved essential in these cases, not only confirming the presence of MetS-related pathology but also offering baseline values for monitoring treatment response. Importantly, emerging research supports the use of inflammatory biomarkers as both diagnostic and prognostic tools in dermatology [26]. A 2023 review underscored that elevated hs-CRP and proinflammatory cytokines (IL-6, TNF-α) correlate with acne severity and systemic comorbidities, particularly in male adolescents with central obesity [27].

Hormonal profiling also proved decisive in the diagnostic workup, especially in the female patient with PCOS and hyperprolactinemia. Beyond its relevance for reproductive health, PCOS represents a common endocrine condition wherein dermatologic signs, inacne, hirsutism, and seborrhea, may precede or dominate the clinical picture. Studies demonstrated that women presenting with acne and menstrual irregularities had a 4-fold increased likelihood of meeting criteria for PCOS, and that early dermatologic recommendation leads to endocrinologic diagnosis in more than 60% of cases [28,29]. These findings strongly support routine androgenic and metabolic screening in young women with persistent acne.

Another important aspect observed in this case series is the therapeutic inertia encountered when systemic contributors are unrecognized. In both male cases, initial resistance to standard antibiotics and topical regimens likely reflected an ongoing pro-inflammatory and insulin-resistant state that perpetuated sebaceous gland activity and follicular occlusion. It was only after metabolic interventions, especially with metformin and dietary changes, that patients began showing consistent improvement. These observations are consistent with data from studies which concluded that adjuvant metformin use in IR-positive acne patients resulted in greater lesion reduction than dermatologic monotherapy alone [30,31].

In addition, treatment decisions were impacted by diagnostic findings. For instance, initiation of isotretinoin was delayed or dose-changed in the context of dyslipidemia and elevated transaminases, as isotretinoin can exacerbate these abnormalities. This highlights the practical value of metabolic workup not only in identifying root causes but also in guiding safe therapeutic choices.

Resistance to conventional dermatologic treatment appeared to mirror the presence of unresolved systemic dysfunctions. In all three patients, partial or limited response to antibiotics and topicals was observed until metabolic or hormonal abnormalities were actively managed. Interventions such as metformin, statins, and structured lifestyle modifications played a crucial role in breaking this resistance, leading to notable improvement in lesion counts and treatment tolerance. Supporting this, cohort studies found that patients with IR had a significantly lower response rate to antibiotics alone, whereas metabolic correction enhanced therapeutic efficacy [32,33].

Inflammation itself emerged as a key therapeutic target, particularly in the third case. Elevated CRP and ASLO titers suggested a systemic inflammatory state beyond classical metabolic dysfunction, pointing to the role of immune activation in driving cutaneous disease. This aligns with growing evidence that pro-inflammatory cytokines and immune dysregulation contribute to acne pathogenesis, particularly in metabolically vulnerable patients [34,35]. Targeting inflammation, whether through metabolic modulation or systemic anti-inflammatory therapy, may therefore represent an essential pillar of treatment in selected cases.

Together, these insights advocate for a paradigm shift in acne management: from a skin-focused model to one that integrates dermatologic, metabolic, and endocrine diagnostics. Recognizing acne as a systemic signal, rather than an isolated cutaneous disorder, enables more personalized, multidisciplinary, and effective therapeutic strategies. Beyond prescriptions, each patient received lifestyle counseling aimed at weight loss, improved insulin sensitivity, and dietary glycemic control, all proven to improve acne severity and metabolic metrics [36]. The interdisciplinary coordination between dermatologists, endocrinologists, and primary care providers allowed for more comprehensive care and better patient engagement [37].

When considering the therapeutic challenges, managing severe acne in the context of MetS poses unique challenges. The early identification of IR and MetS in our patients allowed us to start metabolic intervention—metformin and statin therapy, dietary changes—simultaneously with dermatologic treatments. This precision-based approach reflects an increasingly accepted model in inflammatory dermatology, wherein identifying systemic contributors allows for more tailored and effective therapy. A recent study emphasized that routine metabolic screening in young adults with severe acne led to earlier diagnosis of IR in 34% of cases, allowing for prompt lifestyle and pharmacologic interventions that improved both dermatologic and metabolic outcomes [38].

One important observation from our series is that standard acne therapies yielded slower or less robust responses than typically expected in otherwise healthy young patients. In Case 1 and Case 2, despite appropriate use of oral antibiotics and potent topical regimens, the improvement after 2–3 months was only partial. This contrasts with many clinical trial results where such therapies significantly reduce lesion counts in that timeframe [39,40,41].

The persistent hyperinsulinemia and androgen excess in our patients may have been counteracting the therapeutic effects. For instance, ongoing high insulin levels could continue to drive sebaceous activity and follicular keratinization, making it harder for medications like retinoids to exert their full effect. Similarly, elevated androgen levels (endogenous) might reduce the impact of treatments, as the underlying stimulus for acne formation remains active. There is some evidence in the literature that acne associated with IR can be more refractory to treatment, often requiring additional measures or longer duration to achieve control [42].

Another consideration is the use of certain first-line acne drugs in metabolically unhealthy patients. Oral isotretinoin is the gold-standard for severe nodulocystic acne. However, isotretinoin commonly causes elevations in triglycerides and cholesterol during therapy and can rarely induce changes in glucose metabolism [43,44]. In patients who already have dyslipidemia or borderline glucose control, isotretinoin could theoretically push them into more dangerous clinical situation: acute pancreatitis if triglycerides spike very high, or unmasking hyperglycemia [45]. A case series has suggested that isotretinoin should be used cautiously in patients with metabolic syndrome risk factors, with frequent lipid monitoring [46].

In our series, we delayed isotretinoin initiation for Cases 1 and 2 until we could get their lipids under better control. In Case 3, we proceeded with isotretinoin due to the severity of acne but simultaneously took measures to mitigate risk (metformin introduction, dietary fat restriction, etc.). Fortunately, none of our patients experienced severe lipid spikes on isotretinoin, in part because of dose management and concurrent treatments.

Pharmacologically, metformin stands out as a useful adjunct in these scenarios. Metformin is an insulin sensitizer widely used in type 2 diabetes and in PCOS management. Recent dermatologic studies and meta-analyses have explored metformin for acne, particularly in patients with signs of insulin resistance or hormonal acne. A meta-analysis found that metformin, either alone or as an add-on, was associated with a significant reduction in acne lesion scores in women with PCOS. The mechanism is presumed to be via lowering insulin (and consequently IGF-1 and androgen levels) as well as potential anti-inflammatory effects [47].

In Case 1 (PCOS female) and Case 3 (male with IR), we initiated metformin therapy, and over a few months, both reported a gradual improvement in their acne in parallel with feeling “less hungry” and losing some weight. While we cannot separate the direct effects of metformin on the skin from its systemic metabolic effects, the overall outcome was positive. Metformin was well-tolerated aside from mild gastrointestinal upset initially, and we continued it as it provided broader health benefits (reducing progression to diabetes, addressing PCOS symptoms). A clinical trial found that metformin monotherapy was nearly as effective as oral antibiotics in reducing acne severity in women with signs of insulin resistance [48].

Thus, in patients who have both acne and metabolic issues, metformin serves as a valuable therapeutic choice, addressing the root metabolic problem and indirectly helping resolve acne. The positive aspect highlighted by these cases is that a multi-pronged approach can yield benefits. While purely dermatologic treatment had limitations, the addition of metabolic interventions likely played a role in the improvements. Weight loss, though modest in our patients, can reduce IR and inflammation. Even a 5–10% weight reduction is known to significantly improve metabolic parameters [49,50].

In our cases, each patient who managed to lose a few kilograms noted not only feeling healthier but also noticed their skin getting less oily and acne flaring less intensely. Dietary modification, particularly adopting a low glycemic load diet, is an evidence-based adjunct for acne management. A study demonstrated in a randomized controlled trial that male acne patients on a low-glycemic-load diet had greater acne improvement and enhanced insulin sensitivity compared to those on a standard high-glycemic diet [36].

Drawing on these insights, we offered all three patients personalized dietary counseling, aiming to reduce foods known to promote insulin resistance and systemic inflammation. The recommendations focused on minimizing high-glycemic processed carbohydrates, sugary snacks, and industrial fast food, while encouraging the consumption of vegetables (leafy and cruciferous), legumes, whole grains, lean proteins (poultry, fish, eggs), and healthy fats (olive oil, avocado, walnuts). More detailed guidance was also provided regarding dairy: only low-fat options such as fresh cheese, unsalted “telemea”, or skimmed milk were allowed, limited to three servings per week. Fermented dairy products like plain yogurt, kefir, or buttermilk were preferred, while other dairy products were excluded. Industrial snacks, sweetened beverages, and desserts were discouraged, with exceptions made for homemade juices and small amounts of dark chocolate. High-omega-6 nuts such as cashews, sunflower seeds, and almonds were restricted, but walnuts were allowed in moderation. Fruits were not limited, provided they were fresh and unsweetened. While adherence varied, the patient who followed the diet most consistently (Case 3) also demonstrated the most rapid clinical and metabolic improvement.

Lifestyle changes were also fundamental. All three patients were counseled extensively on diet and exercise, and we collaborated with a nutritionist for Case 1. The improvements in Cases 2 and 3 were likely blunted by the fact that teenage patients often struggle with strict dietary adherence. Nonetheless, even incremental lifestyle improvements can set the stage for better long-term control. It is important for clinicians to continue reinforcing these measures, as the benefits go beyond the skin.

These cases highlight the role of screening and multidisciplinary care. A simple measurement of waist circumference and BMI, as well as basic lab tests (fasting glucose, lipid profile), can unmask a MetS that might otherwise go undiagnosed in a young person. In fact, waist circumference turned out to be an independent predictor of metabolic risk in an acne cohort study [2], emphasizing that a quick tape measure around the patient’s midsection is informative. In our cases, identifying the MetS components allowed for timely referral and co-management with primary care/endocrinology. This kind of interdisciplinary approach ensures that while we treat the acne aggressively, the patient is also being evaluated for prediabetes, hypertension, or dyslipidemia, conditions with implications for future health if left unaddressed. For the patients, this holistic care has the added benefit of demonstrating that we are treating “the whole person,” which can improve adherence to therapy and follow-up.

Viewing severe acne as a potential systemic red flag represents a shift in the traditional paradigm. Acne has long been considered purely an aesthetic or quality-of-life condition; emerging data, including our cases, suggest it can also be a marker of internal health status. This does not mean all acne patients have metabolic syndrome, but it does mean clinicians should remain vigilant for those who do. Particularly, males with nodulocystic acne and concomitant obesity, or females with acne plus menstrual/hormonal issues, merit a metabolic work-up. Early intervention in MetS, through lifestyle or medication, can prevent progression to type 2 diabetes or cardiovascular disease [51]. Treating the patient’s MetS is a form of preventative medicine that the dermatologist can help initiate.

While these cases provide valuable insights into the potential interplay between acne and metabolic dysfunction, they should be interpreted within the methodological limitations of a case series. The small sample size and lack of a control group limit the ability to draw causal conclusions or generalize findings. Moreover, all patients in our series were either overweight or obese according to BMI, which may act as a confounding factor in the observed association. Although the diagnosis of metabolic syndrome followed internationally accepted criteria, including waist circumference and metabolic parameters, we cannot entirely isolate the influence of excess body weight on acne severity. Additionally, the relatively short and heterogeneous follow-up period limits our ability to assess the sustainability of therapeutic outcomes. These findings should therefore be viewed as hypothesis-generating, warranting further investigation in controlled studies with larger samples, BMI stratification, and long-term metabolic monitoring.

## 5. Conclusions

The presented cases illustrate how diagnostic precision, achieved through comprehensive clinical evaluation, targeted laboratory testing, and interdisciplinary collaboration, can enhance therapeutic decision-making in patients with severe or treatment-resistant acne. While causality cannot be established from these observations, the findings suggest that acne may, in selected patients, serve as a cutaneous indicator of underlying metabolic or hormonal dysfunction. Recognizing this broader clinical context may encourage earlier metabolic screening and the adoption of more individualized, long-term treatment strategies.

## Figures and Tables

**Table 1:** Metabolic and endocrine parameters for Case 1.

Parameter	Value	Reference Range *	Interpretation
Fasting glucose	100 mg/dL	70–99 mg/dL	High-normal, prediabetic risk
HbA1c	5.84%	<5.7%	Elevated, suggests early glucose dysregulation
HOMA-IR	2.60	≤2.0	Indicates insulin resistance
Triglycerides	120 mg/dL	<150 mg/dL	Mildly elevated
LDL cholesterol	135 mg/dL	<100 mg/dL (optimal)	Elevated
HDL cholesterol	30 mg/dL	>50 mg/dL (female)	Decreased
Total testosterone	82 ng/dL	20–73 ng/dL	Elevated, consistent with PCOS
Serum prolactin	52.5 ng/mL	1.9–25 ng/mL	Elevated, due to microprolactinoma
BMI	30.4 kg/m^2^	18.5–24.9 kg/m^2^	Obesity, Class I
Waist circumference	98 cm	<88 cm (female)	Visceral obesity

HbA1c, glycated hemoglobin; HOMA-IR, homeostatic model assessment of insulin resistance; LDL, low-density lipoprotein; HDL, high-density lipoprotein; PCOS, polycystic ovary syndrome; TSH, thyroid-stimulating hormone; T4, thyroxine; BMI, body mass index. * Reference ranges are based on the standard values provided by the clinical laboratory of Pelican Hospital, Oradea, Romania, at the time of testing.

**Table 2:** Therapeutic regimen for Case 1.

Treatment Category	Intervention	Clinical Purpose
Systemic dermatologic	Doxycycline 100 mg/day for 3 months	Anti-inflammatory, antimicrobial
Topical dermatologic	Benzoyl peroxide 5% (AM, once daily)	Antibacterial and comedolytic
	Tretinoin 0.05% (PM, three times per week)	Normalizes keratinization
	Azelaic acid 20% (AM, alternate days, four times per week)	For comedowns and PIH
	Clindamycin/erythromycin topical (BID on active lesions)	Reduces local inflammation
	Niacinamide, adapalene-based products, moisturizer (once daily)	Barrier repair, anti-inflammatory
Procedural therapies	Comedown extraction + superficial glycolic acid chemical peels (monthly)	Improve scarring and comedonal load
Hormonal therapy	* COCs (EE + drospirenone)	Suppress ovarian androgens, cycle regulation
	* Cabergoline	Prolactin control

COCs, combined oral contraceptives; EE, ethinyl estradiol; PIH, post-inflammatory hyperpigmentation; BID, twice daily; LDL, low-density lipoprotein; TG, triglycerides. * in accordance with the specialist physician’s recommendation.

**Table 3:** Metabolic and endocrine parameters for Case 2.

Parameter	Value	Reference Range *	Interpretation
Fasting glucose	105 mg/dL	70–99 mg/dL	Elevated—high–normal range
HbA1c	Not measured	<5.7%	Not available
HOMA-IR	3.57	≤2.0	Confirmed insulin resistance
Triglycerides	160 mg/dL	<150 mg/dL	Mildly elevated
LDL cholesterol	139 mg/dL	<100 mg/dL (optimal)	Elevated
HDL cholesterol	35 mg/dL	>50 mg/dL (male)	Decreased
Total testosterone	89 ng/dL	300–1000 ng/dL (age-specific)	Slightly above average, not pathological
Blood pressure	135/85 mmHg	<120/80 mmHg	Prehypertension
BMI	30.2 kg/m^2^	18.5–24.9 kg/m^2^	Obesity, Class I
Waist circumference	98 cm	<94 cm (male)	Central/visceral obesity

HbA1c, glycated hemoglobin; HOMA-IR, homeostatic model assessment of insulin resistance; LDL, low-density lipoprotein; HDL, high-density lipoprotein; BMI, body mass index. * Reference ranges are based on the standard values provided by the clinical laboratory of Pelican Hospital, Oradea, Romania, at the time of testing.

**Table 4:** Therapeutic regimen for Case 2.

Treatment Category	Intervention	Clinical Purpose
Systemic dermatologic	Doxycycline 100 mg/day for 8–12 weeks	Reduce *C. acnes*, inflammation
Topical dermatologic	Clindamycin/erythromycin creams BID	Anti-inflammatory agents for active lesions
	Salicylic acid 2% cleansing gel BID	Keratolytic action; pore unclogging
	Benzoyl peroxide 5% gel (AM, once daily)	Antimicrobial, anti-inflammatory
	Adapalene 0.1% gel (PM, once daily)	Normalization of keratinization
	Tretinoin (intermittently at night (three times per week)	Enhanced comedolysis in dense areas
	Clindamycin solution (AM)/erythromycin gel (PM), alternate days	Local treatment of inflamed papules/pustules
	Non-comedogenic moisturizer (once daily)	Prevent irritation from topical regimen
	Manual comedown extractions (monthly)	Reduce comedonal burden
Lifestyle interventions	Low glycemic index/load diet	Improve IR and acne severity
	Exercise: 150 min/week (moderate intensity)	Weight reduction, metabolic improvement
Metabolic therapy	Atorvastin 10 mg/day	LDL and triglyceride control
	Referral to PCP for dyslipidemia management	Monitor and adjust lipid-lowering therapy

BID, twice daily; AM, morning; PM, evening; IR, insulin resistance; LDL, low-density lipoprotein; PCP, primary care physician.

**Table 5:** Metabolic and endocrine parameters for Case 3.

Parameter	Value	Reference Range *	Interpretation
Fasting glucose	98 mg/dL	70–99 mg/dL	Normal range
HOMA-IR	2.88	≤2.0	Confirmed insulin resistance
Triglycerides	173 mg/dL	<150 mg/dL	Elevated
LDL cholesterol	142 mg/dL	<100 mg/dL (optimal)	Elevated
HDL cholesterol	38 mg/dL	>50 mg/dL (male)	Decreased
High-sensitivity CRP	10 mg/L	<0.6 mg/L	Mild systemic inflammation
ASO titer	226 IU/mL	<200 IU/mL	Elevated, post-streptococcal immune activity
Total testosterone	78 ng/dL	50–75 ng/dL (age-adjusted)	Upper-normal or mildly elevated
BMI	29.4 kg/m^2^	18.5–24.9 kg/m^2^	Overweight (nearing obesity)
Waist circumference	94 cm	<94 cm (male)	Borderline central obesity

HbA1c, glycated hemoglobin; HOMA-IR, homeostatic model assessment of insulin resistance; LDL, low-density lipoprotein; HDL, high-density lipoprotein; CRP, C-reactive protein; ASO, antistreptolysin O; BMI, body mass index. * Reference ranges are based on the standard values provided by the clinical laboratory of Pelican Hospital, Oradea, Romania, at the time of testing.

**Table 6:** Therapeutic regimen for Case 3.

Treatment Category	Intervention	Clinical Purpose
Systemic dermatologic	Minocycline 100 mg/day (initial, 60 day), then switched to Isotretinoin (30 mg/day-6–8 months)	Anti-inflammatory; sebo-suppressive; comedolytic
Topical dermatologic	Adapalene 0.1% (PM), benzoyl peroxide 5% (AM, daily)	Normalize keratinization, reduce bacterial load
	Azelaic acid 20% cream (four times per week)	Anti-inflammatory, depigmenting, comedolytic
	Erythromycin cream (BID on active lesions)	Reduce pustular activity
Metabolic therapy	Metformin 500 mg BID (1000 mg/day)	Improve insulin sensitivity, support weight control
Lifestyle interventions	Low-carb, low glycemic index diet + aerobic/resistance exercise	Reduce IR and systemic inflammation
Monitoring	Liver enzymes, lipids during isotretinoin	Prevent treatment-related metabolic complications

PM, evening; AM, morning; BID, twice daily; IR, insulin resistance.

**Table 7:** Summary comparison of the three acne cases and their multidisciplinary management.

Feature	Case 1	Case 2	Case 3
Age/Sex	23/female	19/male	18/male
Acne duration	>5 years	~2 years	~1 year (rapid progression)
Acne type	Inflammatory moderate–severe	Severe nodulocystic	Severe nodulocystic, early conglobata
Metabolic risk	PCOS, IR, MetS, central obesity, hypercholesterolemia	MetS with IR and dyslipidemia, prehypertension	Dysmetabolic profile, IR, elevated CRP and ASO
BMI/central adiposity	30.4 kg/m^2^ (Class I obesity), WC: 98 cm	30.2 kg/m^2^ (Class I obesity), WC: 98 cm	29.4 kg/m^2^ (overweight), WC: 94 cm
Treatment—systemic	Doxycycline, COCs, spironolactone	Doxycycline, statin	Minocycline (100 mg-60 days → Isotretinoin (30 mg/day), metformin
Treatment—topical	BPO, tretinoin, azelaic acid, ABs	BPO, adapalene, SA cleanser, ABs	BPO, adapalene, azelaic acid, ABs
Metabolic therapy	Lifestyle and dietary adjustments	Lifestyle and dietary adjustments, statin (atorvastatin 10 mg)	Metformin 1000 mg/day, structured diet + exercise
2-month acne response	Partial, reduced inflammation	Modest, slower than expected	Moderate, fewer nodules, weight loss
Weight change	+ slight (1 KG) ↓ at 2 months	~2 kg ↓ by 3 months	~5 kg ↓ by 2 months
HOMA-IR	2.60 → not reassessed at 2 months	3.57 → not reassessed at 2 months	2.88 → 2.18 (↓ at 2 months)
Triglycerides (mg/dL)	120	160 → 120 ↓ at 3 months	173 → 143 (↓ at 2 months)
LDL cholesterol (mg/dL)	135 → 129 ↓ at 2 months	139 → 96 ↓ at 3 months	142 → 125 (↓ at 2 months)
HDL cholesterol (mg/dL)	30 → 36	40	38 → 44 (↑ at 2 months)
CRP (mg/L)	Not measured	Not measured	10 → 3.5 (↓ at 2 months)
Planned escalation	Isotretinoin (pending lipid control)	Isotretinoin (if no further improvement)	Continue isotretinoin full course

IR, insulin resistance; MetS, metabolic syndrome; COCs, combined oral contraceptives; ABs, topical antibiotics; BPO, benzoyl peroxide; SA, salicylic acid; ↑, increase; ↓, decrease; →, change from baseline to follow-up

## Data Availability

The data are available from the first author, per request.

## Abbreviations

The following abbreviations are used in this manuscript:

AM	Morning
ASO	Antistreptolysin O
AST	Aspartate transaminase
BID	Twice daily
BMI	Body mass index
COCs	Combined oral contraceptives
CRP	C-reactive protein
EE	Ethinylestradiol
EWL	Excess weight loss
GAD-7	Generalized anxiety disorder-7
GGT	Gamma-glutamyl transferase
GI	Gastrointestinal
Hb	Hemoglobin
HbA1c	Glycated hemoglobin
HDL	High-density lipoprotein
HOMA-IR	Homeostatic model assessment of insulin resistance
ICD-10	International Classification of Diseases, 10th Revision
IGF-1	Insulin-like growth factor 1
IR	Insulin resistance
IL-6	Interleukin 6
LDL	Low-density lipoprotein
MetS	Metabolic syndrome
mTORC1	Mammalian target of rapamycin complex 1
PCOS	Polycystic ovary syndrome
PCP	Primary care physician
PIH	Post-inflammatory hyperpigmentation
PM	Evening
SA	Salicylic acid
SpO_2_	Peripheral oxygen saturation
TG	Triglycerides
TNF-α	Tumor necrosis factor alpha
TWL	Total weight loss
TSH	Thyroid-stimulating hormone
T4	Thyroxine
